# A Handheld Real-Time Photoacoustic Imaging System for Animal Neurological Disease Models: From Simulation to Realization

**DOI:** 10.3390/s18114081

**Published:** 2018-11-21

**Authors:** Yu-Hang Liu, Yu Xu, Lun-De Liao, Kim Chuan Chan, Nitish V. Thakor

**Affiliations:** 1 Singapore Institute for Neurotechnology (SINAPSE), National University of Singapore, Singapore 117456, Singapore; E0012410@u.nus.edu (Y.X.); ldliao@nhri.org.tw (L.-D.L.); lsickc@nus.edu.sg (K.C.C.); eletnv@nus.edu.sg (N.V.T.); 2 Department of Biomedical Engineering, National University of Singapore, Singapore 117583, Singapore; 3 Institute of Biomedical Engineering and Nanomedicine, National Health Research Institutes, Miaoli 35053, Taiwan; 4 Department of Biomedical Engineering, Johns Hopkins University, Baltimore, MD 21205, USA

**Keywords:** neurological disease, photoacoustic imaging, stroke, tumor, vascular function

## Abstract

This article provides a guide to design and build a handheld, real-time photoacoustic (PA) imaging system from simulation to realization for animal neurological disease models. A pulsed laser and array-based ultrasound (US) platform were utilized to develop the system for evaluating vascular functions in rats with focal ischemia or subcutaneous tumors. To optimize the laser light delivery, finite element (FE)-based simulation models were developed to provide information regarding light propagation and PA wave generation in soft tissues. Besides, simulations were also conducted to evaluate the ideal imaging resolution of the US system. As a result, a PA C-scan image of a designed phantom in 1% Lipofundin was reconstructed with depth information. Performance of the handheld PA system was tested in an animal ischemia model, which revealed that cerebral blood volume (CBV) changes at the cortical surface could be monitored immediately after ischemia induction. Another experiment on subcutaneous tumors showed the anomalous distribution of the total hemoglobin concentration (HbT) and oxygen saturation (SO_2_), while 3D and maximum intensity projection (MIP) PA images of the subcutaneous tumors are also presented in this article. Overall, this system shows promise for monitoring disease progression in vascular functional impairments.

## 1. Introduction

Photoacoustic (PA) imaging is a hybrid imaging technology that provides multiscale spatial resolution and deep tissue penetration by utilizing intrinsic optical absorption with ultrasound (US) detection [1,2,3]. Currently, the development of real-time PA imaging systems for biomedical applications (e.g., breast tumor and melanoma detection, vascular structure imaging and evaluation of muscle oxygenation) is greatly needed to assess anomalous tissue perfusion resulting from conditions such as ischemia or subcutaneous tumors [4,5,6,7,8,9,10]. In addition, lots of novel handheld PA imaging systems have been proposed to provide diagnostic information for preclinical implementation [11,12,13]. Most studies have provided their strategies for developing the handheld PA systems, such as using a customized linear transducer array or hemispherical transducer array to acquire real-time imaging [13]. However, the details of simulation for designing a handheld PA probe have not been explicitly discussed in most articles. Specifically, enough photons need to be delivered and absorbed by the target within the American National Standards Institute (ANSI) safety limit to acquire PA imaging with a high signal-to-noise ratio (SNR) at a deep penetration depth in scattering medium. Therefore, simulations for designing a PA probe are needed.

The random directions of radiation caused by multiscattering and absorption dominate the light propagation in biological tissues, compromising light delivery to selected targets for optical and PA imaging. Therefore, previous research studies have attempted to optimize the designs of PA systems for biomedical applications to increase detection sensitivity and observe targets at a deeper depth [14,15,16]. The radiative transfer equation (RTE) has been commonly employed to simulate light propagation in turbid materials. The Monte Carlo method, based on the RTE, provides a solution for light propagation simulation [17]. Recently, the Monte Carlo method has been used to evaluate the designs of both dark-field and bright-field PA tomography [14]. The researchers analyzed how the beam width and length, incident angle, and interval between illumination sources affect the light distribution in scattering medium [14]. However, the boundary condition of the incident surface and interfaces between different layers of the Monte Carlo model is simplified (i.e., only initial weight is assigned based on the properties of the medium to determine the percentages of reflection and transmission) [18]. In other words, the Monte Carlo simulation may encounter certain events such as the transition exaggeration between different layers [19]. Alternatively, the finite element method (FEM) is another approach for solving the RTE and estimating light propagation in biological tissues. FEM solves problems in a finite geometric domain with the application of appropriate boundary conditions, and a diffusion approximation of RTE is normally applied with FEM when dealing with the forward scattering problem of photons. Previous studies have derived the progression of PA wave generation from the diffusion equation to pressure propagation [20], investigation of PA image reconstruction [21] and simulation of PA effects at the microscopic level [22]. Thus, a complete model based on FEM (e.g., COMSOL) should be included for evaluating both light propagation estimation (i.e., the diffusion equation) and PA signal generation (i.e., heat generation, stress change, and the acoustic wave equation) to investigate the significant factors affecting the generation of PA signals. For instance, Wang et al. designed a new light illumination scheme (light catcher) based on a FEM-based simulation of PA imaging [15]. Thus, FEM simulations were included in this study to provide an optimal way to deliver the light for acquiring PA imaging.

In this study, we will provide a guide to the development of a linear array-based, handheld, real-time PA imaging system designed for neurological applications, along with simulations and experimental results. In our design, the employed laser source with a fast-wavelength-tuning function can generate 100 high-energy pulses per second within the near-infrared (NIR) window (i.e., various wavelengths could be selected for different applications), ensuring the quality of multispectral imaging if multiple chromophores need to be evaluated at different wavelengths. Integration of this laser with a fast data acquisition US platform allows the required information to be analyzed in real-time with a high framerate. First, the results of simulation and phantom to evaluate the performance of the developed system are presented. Next, the system was also utilized to investigate the hemodynamic functions in rodent models of photothrombotic ischemia (PTI) and subcutaneous tumors. This handheld PA imaging system demonstrates real-time observations of symptoms/impairments in vascular functions.

## 2. Materials and Methods

To ensure that enough photons could be delivered and absorbed by the target for generating PA signals with a high SNR, we first need to conduct simulations using multiple models (e.g., heat transfer) to optimize the parameters. Then, after determination of the parameters for light delivery, we could customize a fiber bundle to integrate with the commercial linear-array transducer as the handheld PA imaging probe. Based on the characteristics of the laser source and US platform, we could further perform the multispectral PA imaging using a handheld probe in real-time.

### 2.1. Light Propagation Simulation in Scattering Medium

For the simulation in scattering medium, the target medium was 1% Lipofundin (B. Braun Singapore Pte. Ltd., Singapore), which has optical properties similar to those of organic soft tissue (i.e., µ_a_ = 0.026 cm^−1^; $\mu_{s}^{\prime }$ = 6.31 cm^−1^; at 785 nm) [23]. The dimensions of the target medium were 160 mm × 80 mm × 50 mm in this model. The position of the light sources for calculation was defined as the location where large scattering occurs:

(1) $$Z0=\frac{1}{\mu_{a}+\mu_{s}^{\prime }}$$

(2) $$\mu_{s}^{\prime }=\left(1-g\right)\mu_{s}$$

where $\mu_{a}$ and $\mu_{s}$ are the absorption and scattering coefficient, respectively, while g is an anisotropic coefficient, defined as the average cosine of scattering angles [18]. $\mu_{s}^{\prime }$ is the reduced scattering coefficient. Z0 is the transport mean free path; within the distance of Z0, photons propagate in its original directions with negligible scattering events. To fit the imaging dimensions of the rectangular-shaped US transducer array detector surface, we defined the dimensions of the laser beam as 16.5 mm × 0.8 mm. For highly scattering materials (i.e., $\mu_{a}\ll \mu_{s}$), such as biological tissues and 1% Lipofundin, the light distribution follows the diffusion equations [15,16,24]:

(3) $$\frac{n}{c}\frac{\partial \varphi }{\partial t}+\nabla \cdot \left(-D\nabla \varphi \right)+\mu_{a}\varphi =P_{0}$$

(4) $$D=\frac{1}{3\left(\mu_{s}^{\prime }+\mu_{a}\right)}$$

(5) $$P_{0}=\mu_{s}^{\prime }\frac{W_{p}}{2\pi \sqrt{\pi }\tau_{p}}\exp\left(-\frac{4{\left(t-\tau_{\mathrm{center}}\right)}^{2}}{\tau_{p}^{2}}\right)$$

where n is the refractive index, c is the speed of light in vacuum (3e^8^ m/s), φ is the fluence in $W/{\mathrm{cm}}^{2}$, and $P_{0}$ is the light source. The simulated laser source was a short pulsed, high-energy laser, with Gaussian energy distributed temporally. $W_{p}$, the energy of each pulse, was 20 mJ in our case. Pulse width ($\tau_{p}$) was 5 ns. $\tau_{\mathrm{center}}$ is the time point where the Gaussian pulse reaches its peak value.

The boundary condition assumption was selected based on the materials (e.g., 1% Lipofundin in this study) for our PA imaging system. For the incident surface, a Robin boundary condition is more appropriate [15,16,25]:

(6) $$-D\nabla \varphi_{\vec{n}}=0.5\frac{1-R_{\mathrm{eff}}}{1+R_{\mathrm{eff}}}\varphi$$

(7) $$R_{\mathrm{eff}}=-1.4399n^{-2}+0.7099n^{-1}+0.6681+0.0636n$$

where n is the refractive index of the incident materials (e.g., n = 1.35 for 1% Lipofundin), and R_eff_ is the internal reflection of uniformly diffuse radiation calculated using a curve fit by Egan and Hilgeman (i.e., Equation (7)). To derive the light fluence (φ) within biological specimens, we used the partial differential equation (PDE) modules of COMSOL Multiphysics (COMSOL Inc., Burlington, MA, USA).

### 2.2. Simulation of PA Wave Propagation Using COMSOL

In this study, PA simulation was conducted on a fairly small scale to reduce the considerable computation time. To mimic the biological tissue environment, we used a graphite sphere with a radius of 6 µm as the imaging target, while the surrounding medium was 1% Lipofundin with a radius of 300 µm. To evaluate the PA response, we considered the following physical phenomena:

(i) Light diffusion/propagation in the scattering medium (calculated in the previous section). We used the acquired fluence intensities (based on different illumination angles and intervals) at 10 mm depth as the fluence input for calculating the PA wave generation.
(ii) Heat generation and transfer inside the target and medium. For nanosecond laser pulse radiation, the temperature T was calculated using the following equation [26,27,28]:
  (8) $$C\frac{\partial T}{\partial t}=\nabla k\nabla T+Q$$
  where C and k are the heat capacity and thermal conductivity, respectively. ρ is the density of the selected material. Q is the heat source, and it can be represented by the absorbed fluence times a yield Y (i.e., Q = ϕμ_a_Y).
(iii) Target stress-strain distribution. We also considered how the strain and stress of the target would change due to the transient temperature rise. The solid stress-strain model was used to evaluate this thermal expansion characteristic [15,27]:
  (9) $$\varepsilon =\beta \left(T_{\mathrm{ref}}-T\right)$$
  where ɛ is the changed strain, and β represents the thermal expansion coefficient. T_ref_ is the reference temperature. The mechanical properties of the material were also included as inputs for this calculation, such as Young’s modulus and Poisson’s ratio.
(iv) Thermal expansion and PA wave generation/propagation. Thermal expansion was modeled using the boundary acceleration between the target and surrounding medium. The dominant equation is shown below:
  (10) $$\vec{n}\cdot \left(\frac{1}{\rho }\nabla p\right)=-\vec{n}\cdot \frac{\partial^{2}\vec{u}}{\partial t^{2}}$$
  where p is the response PA wave. The propagation of the PA wave in a medium follows the equation [15,22,27]:
  (11) $$\frac{1}{\rho c_{s}^{2}}\frac{\partial^{2}p}{\partial t^{2}}-\nabla \cdot \left(\frac{1}{\rho }\nabla p\right)=0$$
  where c_s_ is the sound velocity [16,27]. Based on the above equations, we could simulate a complete procedure of PA wave generation for evaluating the proposed strategies of the PA imaging system design. Note that a schematic of simulation procedures using the FEM method is shown in Figure S1 (Supplementary Materials) to illustrate the simulation strategy in details.

### 2.3. Handheld, Real-Time Photoacoustic Imaging System

**Figure 1:**
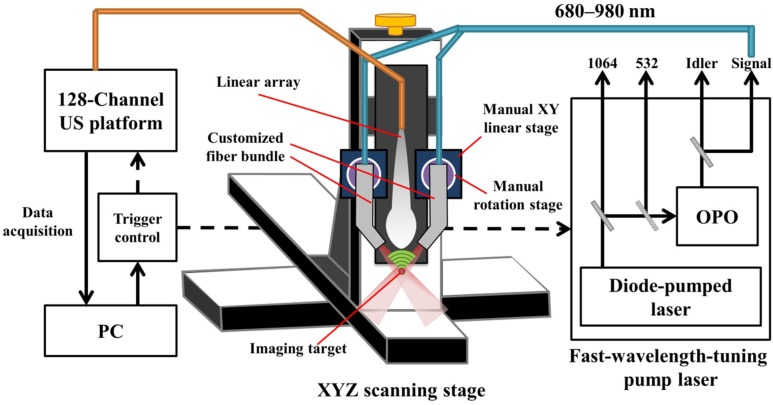
The handheld, real-time photoacoustic (PA) imaging system. One custom-designed optical parametric oscillator (OPO) diode-pumped by a Nd:YAG laser at 532 nm was employed for laser illumination with a fast-wavelength-tuning function for each pulse. The customized fiber bundle was used to deliver the laser light onto the target. Light was evenly distributed into the two arms with a rectangular output size of 16.5 mm × 0.8 mm. A 128-channel research ultrasound (US) platform with a high-frequency array transducer was used for recording the generated PA signal. For proof of simulation results, the PA probe (i.e., fiber bundle with US transducer array) was mounted on the linear and rotation stages to adjust the interval and angle of the two arms. For phantom and in vivo studies (e.g., small animal experiments), the XYZ 3-axis scanning stage with frame holder was used to precisely/automatically image the region of interest (ROI) of the target in either the X or Y direction.

In this study, we simulated, designed and developed a handheld, real-time PA imaging system with a fast multispectral function, as shown in Figure 1. An optical parametric oscillator (OPO) (SpitLight EVO 200 OPO, InnoLas Laser GmbH, Krailling, Germany; 60 × 30 × 9 cm^3^, 30 kg) was employed for laser illumination, while the input laser light was generated by a diode-pumped neodymium-doped yttrium aluminum garnet (Nd:YAG) laser at 532 nm to achieve a high signal output energy (27 mJ to 35 mJ based on the wavelength selection). The tuning wavelength range of the signal output is from 680 to 980 nm in the NIR region for deep penetration depth in biological tissues, while the wavelength range from 1180 to 2400 nm can also be provided by the Idler output port. This OPO can tune the wavelength per pulse at a 100-Hz repetition rate (i.e., the wavelength shift time is less than 10 ms, full span) to achieve the goal of fast multispectral functionality. In this study, we used only 800 nm for cerebral blood volume (CBV) evaluation, and 750 nm/850 nm for acquiring HbT and SO_2_ information in real-time. The pulse width of the laser light from the OPO is 4–7 ns (suitable to generate the PA signal), while the line width is 10–450 cm^−1^. Light from the OPO output was coupled with a customized fiber bundle (CeramOptec GmbH, Bonn, Germany) comprising 597 fibers. The input connector of the fiber bundle was specifically designed based on the dimensions of the output socket of the OPO; thus, the fiber bundle can be directly inserted into the OPO to decrease the laser energy loss. The fiber bundle has the following outputs: one single fiber was designed with a subminiature version A (SMA) connector for monitoring fluctuations in the laser energy; the remaining fibers were evenly distributed into two other arms with a rectangular output size of 16.5 mm × 0.8 mm. Note that a schematic of the customized fiber bundle design is shown in Figure S2 (Supplementary Materials).

The generated PA signal was detected by a high-frequency US transducer array (L22-14v, Verasonics Inc., Kirkland, WA, USA) designed for imaging fine structures. This array is a 128-element linear transducer array with −6 dB bandwidth of 67% and center frequency at 18.5 MHz, while the pitch of each element is 0.10 mm. A 128-channel research US platform (Vantage 128, Verasonics Inc.; 50 × 30 × 50 cm^3^, 35 kg) was used for recording the PA signal. For performing high-quality, real-time imaging, 14-bit analog-to-digital (A/D) converters were included in the platform with a sample rate up to 62.5 MHz. For phantom and in vivo studies (e.g., small animal experiments), a customized XYZ 3-axis scanning stage (LS-110 with Hydra and Pollux controllers, Physik Instrumente (PI) GmbH & Co, Karlsruhe, Germany) with a frame holder was used to precisely image the region of interest (ROI). The travel range of each axis is 102 mm, and the step size can be as low as 1 µm, which can cover the entire region of a small animal’s cortex or hind leg with adequate accuracy. The total cost of the developed PA imaging system (including the laser source, US platform, scanning stage and the fiber bundle) is approximately $SGD 380 K.

When testing the system setup with phantoms (e.g., pencil lead and thin hair in a water tank filled with 1% Lipofundin), the arms of the fiber bundle were mounted on two manual linear stages (PMT2, PHOTONIK, Singapore) and rotation stages (MSRP01/M, Thorlabs, Newton, NJ, USA), which were fixed on the Z-axis of the scanning stage. The manual linear stages can adjust the interval between the two arms and ensure that these two arms are at the same height, while the rotation stage can adjust the light incident angle to verify the simulated results. In addition, the Z-axis of the scanning stage can adjust the illumination depth of the excitation beams. Next, the linear-array US transducer was fixed between the two arms of the fiber bundle for recording the generated PA signal, as shown in Figure 1. The experimental setup for PA imaging of small animals is similar to the setup of the phantom experiments. During the imaging process, both the US transducer array and the output arms of the fiber bundle were immersed in an acrylic water tank with a square hole at the bottom, which was sealed with 15-µm-thick polyethylene film. A thin layer of US gel was applied between the thin film and the small animal’s organ, such as the skin (for tumor imaging) or cortex (for stroke imaging), to ensure proper acoustic coupling for in vivo experiments. The scanning stage was used to scan the ROI of the target in either the X or Y directions. The PA signal recorded by the US transducer was further processed by the US platform to perform real-time PA imaging. 

One optimized wavelength (i.e., 800 nm) was employed to monitor the changes in CBV. The CBV was assumed to be proportional to the specific cortical region at 800 nm (very close to the isosbestic point where the absorption of oxygenated and deoxygenated hemoglobin is identical) [29]. In addition, the total hemoglobin concentration (HbT) and oxygen saturation (SO_2_) were estimated by using two wavelengths (i.e., 750 nm and 850 nm), based on the optical absorption coefficient of hemoglobin at different wavelengths [29]:

(12) $$\mu_{a}^{\lambda }=\varepsilon_{{\mathrm{HbO}}_{2}}^{\lambda }[{\mathrm{HbO}}_{2}]+\varepsilon_{\mathrm{Hb}}^{\lambda }[\mathrm{Hb}]$$

(13) $$\mathrm{HbT}=\left[{\mathrm{HbO}}_{2}\right]+[\mathrm{Hb}]$$

(14) $$O_{2}=\frac{\left[{\mathrm{HbO}}_{2}\right]}{\left[{\mathrm{HbO}}_{2}\right]+[\mathrm{Hb}]}$$

where µ_a_ is the optical absorption coefficient; ε_Hb_ and ε_HbO2_ are the molar extinction coefficients of Hb and HbO_2_, respectively; and [Hb] and [HbO_2_] are the molar concentrations of Hb and HbO_2_, respectively [29,30]. For the maximum intensity projection (MIP) calculation, multiple B-scan images were first collected to form a 3D data set. Afterwards, a specific viewpoint (e.g., X-Y plane) was selected, and the image was reconstructed based on the pixels with maximum intensities of each column (e.g., the A-scan line along the Z-direction) covering the ROI. 

### 2.4. Animal Experiments

All animal experimental protocols were approved by the Institutional Animal Care and Use Committee (IACUC) of the National University of Singapore (NUS). We also confirmed that all methods were performed in accordance with the guidelines and regulations of IACUC and the Office of Safety, Health & Environment (OSHE) of NUS. Animals were housed at a constant temperature and humidity with free access to food and water. For the stroke study, three adult male Sprague-Dawley (S-D) rats were used for the experiments. For PTI induction [2], the photosensitizer Rose Bengal (Sigma, St. Louis, MO, USA) was diluted to 10 mg/mL in 4-(2-hydroxyethyl)-1-piperazineethanesulfonic acid (HEPES)-buffered saline and injected into the tail vein at 0.2 mL/100 g rat body weight. Then, a selected cortical blood vessel within a drilled cranial window (8 × 6 mm^2^) was illuminated using a 5 mW, 532 nm continuous-wave (CW) laser light (AMGA-005, ONSET, Taipei, Taiwan) to generate an occlusion. PA imaging was conducted pre- and post-PTI to evaluate CBV changes. For the subcutaneous tumor study, 4T1 mammary carcinoma cells were cultured *in vitro* before injection into immunocompromised mice. Tumor cells were inoculated subcutaneously at 1 × 10^5^ viable cells in the right hind leg, and the tumor region was imaged using our PA imaging system 7 days after inoculation. Total three female NCr-nude mice were imaged for this study. Further details regarding animal preparation have been previously reported [2,31,32]. Note that all data generated or analyzed during this study are included in this article (and its Supplementary Materials file).

## 3. Results

### 3.1. Simulation Results—Ultrasound Response Based on Vantage Software

The point spread function (PSF), the response of an imaging system to a point source, is an indicator for evaluating the performance (e.g., the spatial resolution) of an imaging system. In this section, we used Vantage software (Verasonics Inc., Kirkland, WA, USA) to simulate the US response for the resolution estimation. Vantage software allows users to define each ideal point scatter (i.e., the dimension is infinitely small) with the following entries: X, Y and Z coordinates and the reflection coefficient. Users can also define a group of scattered points close enough for creating a clutter in geometry. Here, we tested the PSF in the imaging area within water. Therefore, we defined only one single scatter at each time point and made this scatter sweep through different positions to cover the entire imaging area.

**Figure 2:**
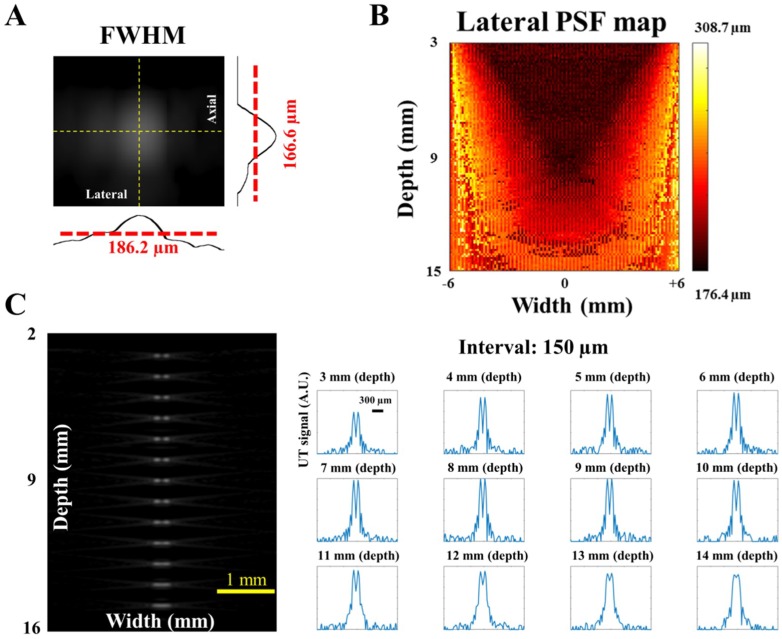
Simulation of the ultrasound (US) response using Vantage software. (**A**) The simulated spatial resolutions of one ideal single point scatter at 10-mm depth and the center of the detecting area. The full width at half maximum (FWHM) of this scatter image was calculated as 166.6 μm (axial) and 186.2 μm (lateral). (**B**) The lateral point spread function (PSF) map of US imaging. The ideal scatter was swept through the entire imaging area (i.e., width: −6 to +6 mm; depth: 3 to 15 mm). The lateral PSF response ranged from 176.4 to 308.7 μm. The imaging resolution decreased when the scatter was at deeper or edge regions. Thus, enough light should be delivered to the depth of 11 mm to acquire PA imaging with consistent imaging quality. (**C**) Two ideal single point scatters were placed in the imaging area with different intervals to assess the lateral resolution. The two scatters were still distinguishable at a depth of 9 mm when the interval between the two scatters was 150 µm. Note that the scale bar in the 3 mm (depth) subfigure is also applied to other subfigures shown in (**C**).

In Figure 2A, an image of the ideal point scatter is shown and analyzed. We measured the full width at half maximum (FWHM) of this image to acquire the lateral and axial PSF responses. For example, the estimated spatial resolutions of the center part of the transducer array at a depth of 10 mm were 166.6 μm (axial) and 186.2 μm (lateral). As mentioned, the ideal scatter was swept through the entire imaging area (i.e., width: −6 to +6 mm; depth: 3 to 15 mm beneath the surface of transducer elements). Both axial and lateral PSF maps of US imaging were calculated accordingly. Based on the physical principles of US, the axial resolution is slightly affected by the imaging depth. Our simulation results showed that the axial resolution of the entire imaging area ranged from 157.2 to 171.6 μm only, which is consistent with the physical principle (i.e., axial resolution = spatial pulse length/2). Note that the differences between the axial resolutions of the entire imaging area were due to the restrictions of the linear-array US transducer structure. Moreover, the imaging depth and the position of point scatter greatly affected the lateral PSF response, mainly due to the beam width spread. As shown in the map of Figure 2B, the lateral PSF response ranged from 176.4 to 308.7 μm. The imaging resolution decreased when the scatter shifted to deeper regions, while the edge regions of the imaging area also exhibited compromised resolution.

Next, an additional method for further determining the spatial resolution was employed. As shown in the left column of Figure 2C, two ideal single point scatters were placed in the imaging area with different intervals (i.e., separations ranged from 100 to 400 μm with a 10-μm step size). The two scatters could not be differentiated when the interval was only 100 μm at any depth. As the interval between the two scatters increased, the two peaks became distinguishable. For instance, the scatters were differentiable at imaging depths shallower than 9 mm when the interval was 150 µm. In addition, the two scatters could be clearly observed at a depth of 15 mm when the interval was 190 μm. According to the above results, the US system should be able to provide high-quality US imaging with approximately 170 μm axial resolution and 190 μm lateral resolution covering the entire imaging area up to a depth of 15 mm. The simulation results provided the characteristic information of the current US imaging system, which is essential for us to design the handheld probe of the PA imaging system. In other words, according to the results of US systemic performance (e.g., Figure 2B), the US transducer and platform can provide an image with stable quality, especially at a depth shallower than 11 mm. That is, for PA imaging, enough laser light should be optimized/delivered to the depth of 11 mm, ensuring a consistent imaging resolution of the target.

### 3.2. Simulation Results—Light Propagation in Scattering Medium

The light propagation of a laser with a bifurcated fiber bundle was evaluated using COMSOL Multiphysics (COMSOL Inc.), a commercial software based on the FEM. The light transmission was discussed under the following conditions: a highly scattering material (in vivo) and water (in vitro). Please refer to the Supplementary Materials for details of light propagation simulation in water.

**Figure 3:**
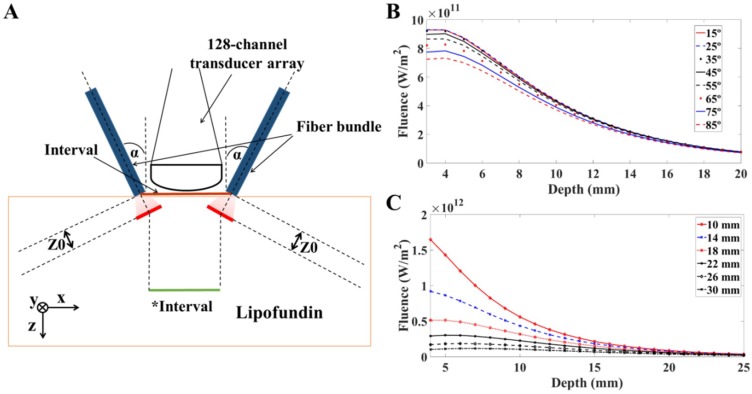
Schematic and simulation results of fluence in 1% Lipofundin. The simulated laser source was a short pulsed (5 ns pulse width), high-energy (20 mJ per pulse) laser, with Gaussian energy distributed temporally. The output beam dimensions of each fiber bundle arm were 16.5 mm × 0.8 mm. (**A**) The simulation configuration for light transmission in 1% Lipofundin. α is the incident angle of the light sources. Z0 is the transport mean free path. Within the distance of Z0, photons propagate in their original directions with negligible scattering events. The Interval is the distance between the two arms of fiber bundle, while the *Interval is the distance between the central points of the red lines (i.e., the light starts to propagate in the diffusive regime). The detector is a 128-channel transducer array. The interval and incident angle are the two main factors included for evaluation. (**B**) The changes in light fluence with respect to different incident angles. The interval was fixed at 14 mm. The light fluence decreased with increasing incident angles. However, the differences in light fluence were minimal when the angle ranged from 15 degrees to 35 degrees. (**C**) The changes in light fluence with respect to different intervals between the two arms of the fiber bundle. The incident angle was fixed at 35 degrees. The fluence value decreased dramatically with an increasing interval from 10 mm to 30 mm. The fluence difference was also reduced as the light propagated deeper into the medium. The results indicated that two arms need to be placed as close together as possible to reach a higher fluence value.

The light illumination model was composed of a highly scattering medium with dimensions of 160 mm × 80 mm × 50 mm and two light illumination sources at the distance of Z0 from the incident surface, as shown in Figure 3A. In our study, the dimensions of the output for each fiber arm were 16.5 mm × 0.8 mm, which were determined by the corresponding length and width of the linear US transducer array. Here, 1% Lipofundin was used as our target medium, and Z0 was calculated based on Equation (1) [33]. After propagating through the distance of Z0, light started to scatter in random directions in the medium. The numerical aperture of the fiber bundle is 0.22 based on the information provided by the fiber bundle manufacturer. Considering the divergence of light, the illumination surface after Z0 would be 16.76 mm × 1.06 mm in 1% Lipofundin. In addition, the reflection rate over human skin ranges from 4% to 7% with variation in the incident angles [34]. Hence, we fixed the reflection rate at 5% for all angles. The observation points for fluence measurement were selected from the midpoints of the interval (Figure 3A) at different depths. According to a previous study, the two main factors influencing the light distribution are the incident angle and the interval between the two fiber arms [14]. Therefore, in this study, we mainly analyzed the fluence change based on these two factors. 

Figure 3B,C were obtained from COMSOL simulation results. We conducted the transient simulation for light fluence distribution, and the observation points were placed along z-axis from the surface of transducer up to the depth of 20 mm. The data was collected and plotted using MATLAB with the variations of angles and intervals. Figure 3B shows the light fluence distribution at different depths with respect to incident angles varying from 15 degrees to 85 degrees. In this study, the interval was maintained at 14 mm, which was the minimum distance between two fiber arms clipped tightly to the US transducer array. The light fluence only slightly changed with the angle ranging from 15 degrees to 35 degrees. When the angle was larger than 35 degrees, the fluence value started to decrease at shallower depths. In addition, the fluence difference between various incident angles gradually decreased as the light propagated to a deeper region. For example, the fluence of 85 degrees at a depth of 3 mm from the surface was approximately 24% less than the fluence with a 15-degree incident angle. In contrast, the difference became only 14% at a depth of 11 mm, while the difference was further reduced at a depth of 20 mm. Figure 3C shows the absolute fluence value change with the interval varying from 10 mm to 30 mm, while the incident angle was fixed at 35 degrees (Figure 3B). When increasing the interval, the absolute fluence value decreased accordingly at all imaging depths. For instance, with a 30-mm interval at a depth of 10 mm, the absolute value of fluence was approximately 18.8% of the value acquired by a 10-mm interval at the same depth. That is, two arms need to be placed as close together as possible to reach a higher fluence value. In addition, the fluence difference was reduced as the light propagated deeper into the medium, but it was still differentiable at depths shallower than 20 mm. Note that the *Interval is the distance between two arms at Z0 locations calculated based on the ballistic photon propagation theory for simulations, as shown in Figure 3A.

Comparing the effects of the incident angle and interval between the two arms of the fiber bundle, in our study, the most essential factor affecting light fluence propagation in scattering medium is the interval. To reach the shortest interval between the fiber arms for practical experiments, we fixed the fiber arms with the transducer array as close as possible and confirmed that the illumination surfaces were on the same plane as the US transducer array (Figure 3A). In this case, the interval was approximately 14 mm (due to the restriction of the US transducer case dimensions) for further verifications of PA wave simulation and phantom experiments.

### 3.3. Simulation Results—PA Wave Propagation in Scattering Medium

**Figure 4:**
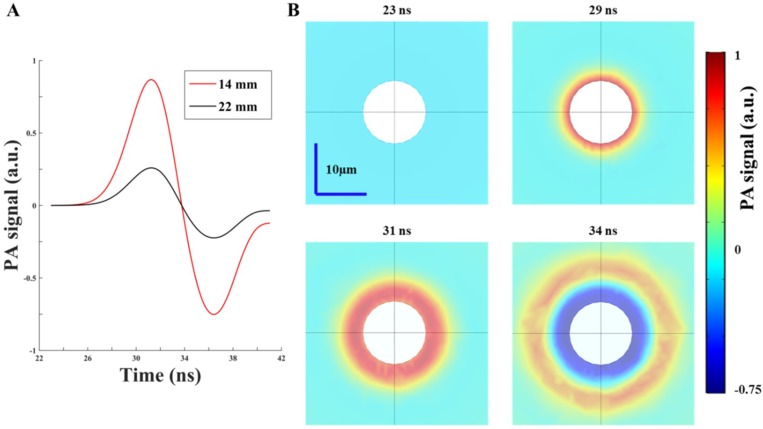
Simulated photoacoustic (PA) response using COMSOL. (**A**) The simulated PA signals with different intervals between the two arms of the fiber bundle. The intensity of the PA signal with a 14-mm interval was 3.35-fold larger than the PA signal with a 22-mm interval. (**B**) The X-Z plane time sequence snapshots of the PA wave field at 4 representative time points in 1% Lipofundin. When the target received a Gaussian light pulse (peak time at 30 ns), the PA wave started to generate from the surface of the target and propagated outward in the scattering medium.

The PA response was simulated in this section using COMSOL. The results of fluence intensity in scattering medium at a depth of 10 mm from the previous section were employed as the input for the PA wave calculation. A graphite sphere with a radius of 6 µm was set as the target and placed into a 1% Lipofundin medium sphere with a radius of 300 µm. An observation point at 1 µm away from the target surface was selected to evaluate the generated PA signal. From the selected observation point, Figure 4A shows the simulated/normalized PA signals with different intervals between the two arms of fiber bundle. The intensity of the PA signal with a 14-mm interval was 3.35-fold of the PA signal with a 22-mm interval, which is consistent with the simulated fluence response in scattering medium (Figure 3C). Note that the Gaussian light pulse achieves the peak value at 30 ns. Figure 4B shows the X-Z plane time sequence snapshots of the PA wave field at 4 representative time points in 1% Lipofundin. When the target received the Gaussian light pulse, the PA wave was generated from the surface of the target and propagated outward in the scattering medium. The simulated PA wave response strongly corresponded to the assigned parameters, such as the laser pulse width (5 ns) and peak time (30 ns), demonstrating the capability of this PA model for performing further PA-related simulations and evaluations. 

### 3.4. Evaluation of Fluence Changes in 1% Lipofundin at Different Depths

**Figure 5:**
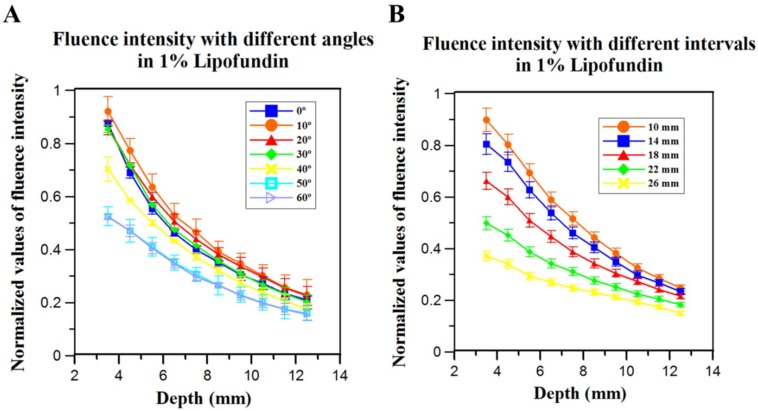
Evaluation of the fluence intensity change in 1% Lipofundin at different depths. (**A**) The angle changed from 0 degree to 60 degrees, while the interval between the two output arms was fixed at 14 mm. (**B**) The interval ranged from 10 mm to 26 mm, while the angle was fixed at 35 degrees for the fluence evaluation. According to (**A**) and (**B**), the angles of the fiber bundle would not largely affect the fluence intensity, while a shorter interval (separation) of the two arms could greatly influence the intensity in scattering medium. Thus, we designed the interval of the two arms to be as short as possible for the system, while the angle was chosen as 35 degrees for experiments in both water and scattering medium.

To verify the simulated fluence results of COMSOL, we used linear and rotation stages in this section for evaluating the relationship between laser intensity with different intervals (and angles) in 1% Lipofundin, while a traditional rectangular shaped fiber bundle (case dimensions of the output arm: 80 × 10 × 40 mm^3^) was employed. The wavelength of the laser light was 785 nm. We poured 1% Lipofundin into the water tank with a square hole at the bottom, which was sealed with 15-µm-thick polyethylene film. A power meter was placed directly under the thin film to measure the fluence intensity. The imaging depth could be precisely adjusted by using the Z-axis of the scanning stage. According to the simulations, the interval between the two output arms was fixed at 14 mm to assess the effect of the angle change solely. The laser intensity dropped at deeper regions for all angles. For example, the laser intensity dropped from 87.5% to 23.5% of the baseline when the imaging depth shifted from 3.5 mm to 11.5 mm for 10 degrees. The intensity differences of different angles were negligible at deeper regions (e.g., 10.5-mm depth). However, there was a significant gap in the laser intensity between 30 degrees and 40 degrees at shallower regions (e.g., 85.5% for 30 degrees and 70.3% for 40 degrees at a depth of 3.5 mm), as shown in Figure 5A. In addition, based on the simulation results of light propagation in water (Figure S3), the incident angles from 35 degrees to 45 degrees would provide quality results for *in vitro* studies. Thus, to integrate the results of both in vivo and *in vitro* experiments, we chose 35 degrees as the light incident angle for the real-time PA system design in this study. 

**Figure 6:**
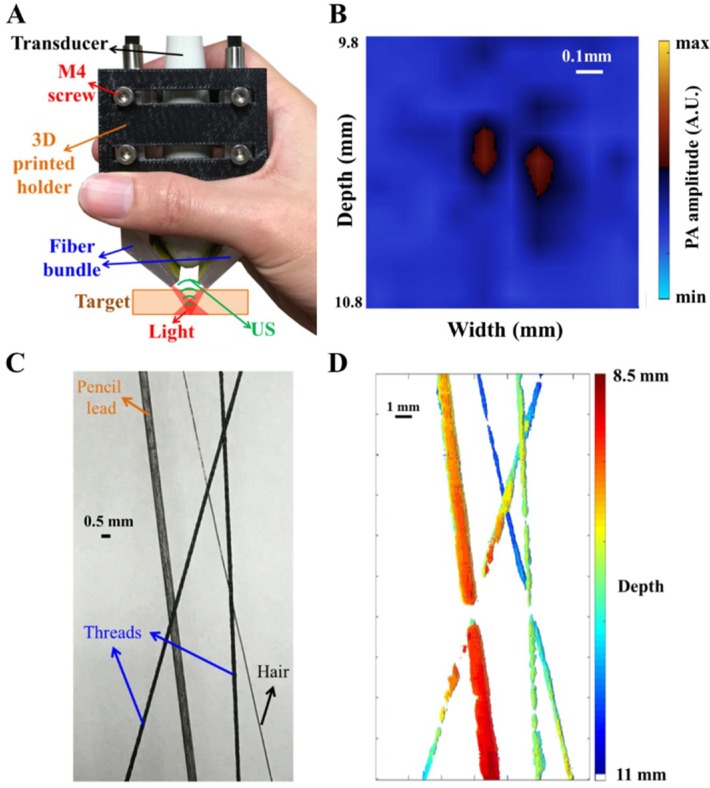
Photoacoustic (PA) image assessment based on the phantom targets. (**A**,**B**) The proof of spatial resolution of the PA imaging system using two thin hairs in 1% Lipofundin. A 3D-printed holder with M4 screws in (**A**) could be used to fix the fiber bundle and transducer array on the scanning stage or directly used for handheld applications. The dimensions of the handheld probe are 4 cm × 5 cm × 8 cm. (**C**,**D**) A photo and color-coded PA image of the designed phantom with depth information. A photo of the designed phantom is shown in (**C**). The scanning stage was used to scan the entire phantom with a 50-µm step size in the Y direction for acquiring multiple B-scan images, and these images were then reconstructed for the C-scan image as shown in (**D**). Note that the interrupted image at the cross point of the pencil lead and thread occurred at a shallower depth (shallower than 8.5 mm). Here, we show the reconstructed PA image between 8.5 mm to 11 mm only due to the dark-field illumination scheme of this PA imaging system.

The influence of the interval between two arms was also evaluated. The angle was fixed at 35 degrees for the fluence assessment. The laser intensity dropped significantly when the interval increased. At a depth of 8.5 mm, the intensity was 44.2% of the baseline for a 10-mm interval compared to 23.1% of the baseline for a 26-mm interval, as shown in Figure 5B. Therefore, the interval should be as short as possible to yield a high fluence, which is consistent with our simulation results (Figure 3C). Due to the restricted case dimensions of the US linear-array transducer, the interval was chosen at 14 mm (the minimal feasible separation) for developing the handheld PA imaging system. Due to the geometry restriction of the two rectangular shaped arms of fiber bundle, we further customized a new fiber bundle (with 35 degrees tilted angle and reduced thickness of output arms) based on the simulation results for experiments, as shown in Figure 6A.

### 3.5. PA Image Assessment Based on Phantom Targets

PA images of the phantom targets are demonstrated in this section. A photo of the PA probe, including the customized fiber bundle and US linear transducer array, is shown in Figure 6A. The M4 screws could be used to fix the fiber bundle on the scanning stage or be integrated with a 3D-printed holder for handheld applications. To examine the spatial resolution of this designed PA system, we placed two thin hairs (~150 µm in diameter) in 1% Lipofundin with a 180-µm interval at a depth of 10.2 mm for evaluation. The pump laser light had a wavelength of 680 nm and an energy density of 10 mJ/cm^2^. Figure 6B shows that PA images of these two thin hairs could be clearly differentiated with a calculated SNR = 11.32 dB, which is consistent with the Vantage simulation of spatial resolution.

In addition, Figure 6C shows a photo of the designed phantom, consisting of one pencil lead (500 µm in diameter), two black threads (~250 µm in diameter) and one black hair (~150 µm in diameter). The entire phantom was also placed in 1% Lipofundin to assess the performance of the PA system. The scanning stage was used to scan the entire phantom for 35 mm in length with a 50-µm step size in the Y direction to acquire multiple X-Z plane B-scan images. Then, these images were reconstructed for the PA C-scan image, as shown in Figure 6D. The results indicated that a color-coded PA image for depth information could be reconstructed, supporting the capability of this PA system. Please refer to Figure S4 of the Supplementary Materials for the results of an additional phantom experiment (including the TopView/SideView MIP and 3D reconstructed PA images).

### 3.6. Performance Evaluation of the PA Imaging System Based on the PTI Model

**Figure 7:**
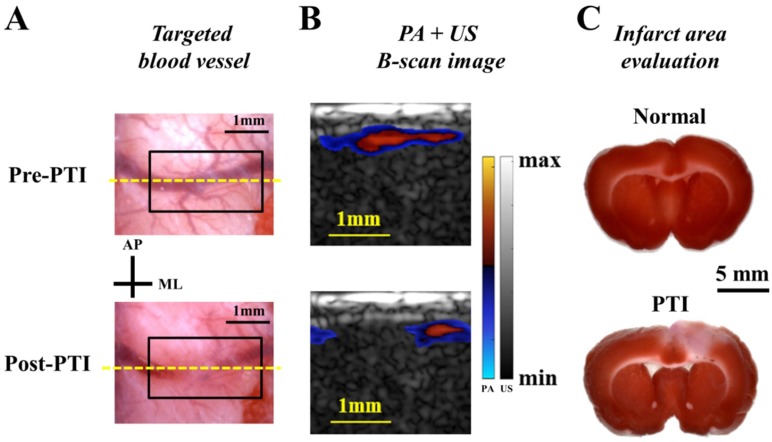
Performance evaluation of the photoacoustic (PA) imaging system based on the PTI model. (**A**) The photos of the selected cortical blood vessel pre- and post-PTI within the cranial window. The yellow dashed line indicates the location of the PA B-scan image, while the black rectangle is the ischemic region. (**B**) Co-registered PA-US B-scan images. The cerebral blood volume (CBV) of the selected cerebral blood vessel shown in the post-PTI image was substantially lower than that shown in the pre-PTI image. (**C**) TTC staining results with and without PTI are presented to support the results of the in vivo PA image.

In vivo experiments were also conducted in this study. A cranial window was created for both ischemic stroke induction and PA imaging [2]. The PTI model was utilized to evaluate the performance of the developed PA system based on changes in the CBV pre- and post-ischemia at 800 nm (very close to the isosbestic point where the absorption of oxygenated and deoxygenated hemoglobin is identical) [29]. In Figure 7A, representative photos of the targeted blood vessel over the right hemisphere pre- and post-PTI are shown. The yellow dashed line in Figure 7A labels the position of the PA B-scan image, while the black rectangle indicates the ischemic region. Corresponding PA B-scan images can be acquired in real-time to evaluate CBV changes in the selected blood vessel. As shown in Figure 7B, the PA images are co-registered with the US image to identify the location of the blood vessels at the cortical surface. Figure 7B shows that the CBV was largely decreased in the target region post-PTI, which is consistent with the photos in Figure 7A. In Figure 7C, the infarct volumes of normal and PTI brains, which are identified as pale (i.e., unstained) in the coronal sections, were also compared using triphenyltetrazolium chloride (TTC) staining at 24 h after the induction of ischemia. The infarct volume results confirmed that ischemia was induced in the rat brain, which was observed by the current PA system during the hyperacute phase of ischemia.

### 3.7. Performance Evaluation of the PA Imaging System Based on the Subcutaneous Tumor Model

**Figure 8:**
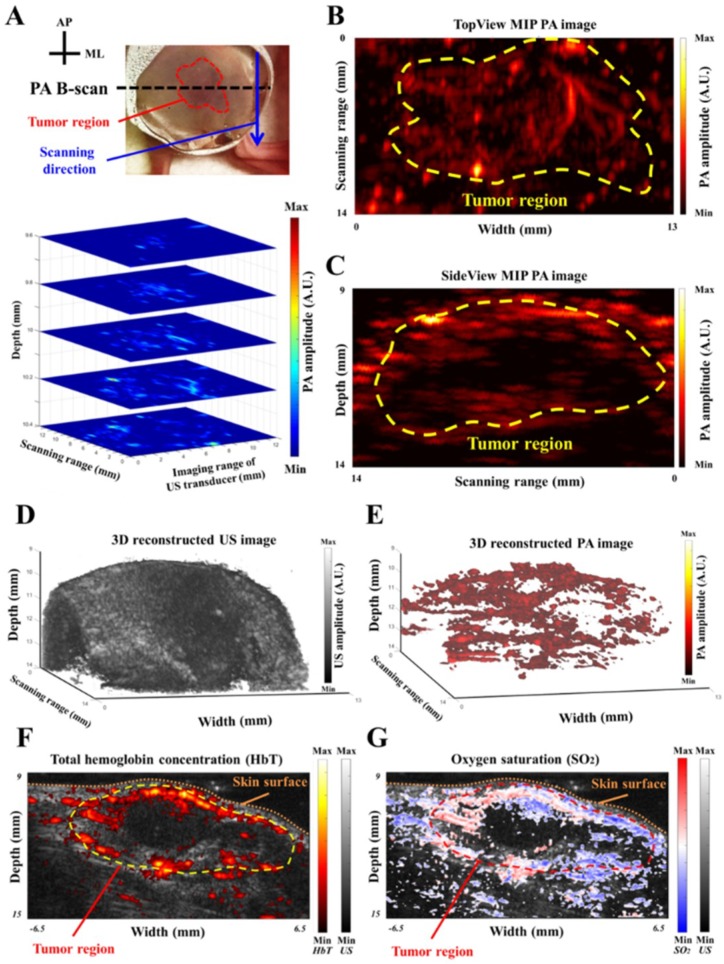
Subcutaneous tumor evaluation using the developed PA imaging system. (**A**) The tumor in the right hind leg was imaged by our PA imaging system. The upper subfigure shows that the tumor region (indicated by red dashed line) was scanned in the scanning direction (blue arrow) to acquire multiple X-Z plane PA B-scan images. The lower subfigure demonstrates the reconstructed 5-slice CBV C-scan images in the X-Y plane at different depths. (**B**,**C**) The TopView (X-Y plane) and SideView (Y-Z plane) MIP images of the subcutaneous tumor, respectively. Tumor regions are indicated by yellow dashed lines. (**D**,**E**) 3D reconstructed US and PA images of the subcutaneous tumor, respectively. (**F**) A normalized total hemoglobin concentration (HbT)-ultrasound (US) co-registered X-Z plane B-scan image. The distribution of HbT is highly correlated with the subcutaneous tumor profile acquired by US imaging. (**G**) A normalized oxygen saturation (SO_2_) image of the subcutaneous tumor. Note that the yellow and red dashed lines indicate the tumor regions in (**F**) and (**G**), respectively.

The subcutaneous tumor model was employed to examine the performance of the developed PA imaging system. Tumor cells were subcutaneously inoculated in the right hind leg, and the tumor region was imaged by our system on Day 7 after inoculation. In the upper subfigure of Figure 8A, the tumor region (indicated by the red dashed line) was scanned for a length of 14 mm with a 50-µm step size in the scanning direction (blue arrow) to acquire multiple X-Z plane real-time PA B-scan images. CBV information for the subcutaneous tumor was acquired at 800 nm. The 5-slice X-Y plane PA C-scan images at different depths were reconstructed immediately after every scanning within 5 s to evaluate the CBV of the subcutaneous tumor caused by angiogenesis, as shown in the lower subfigure of Figure 8A. Besides, TopView (Figure 8B) and SideView (Figure 8C) MIP PA images of the subcutaneous tumor were also concurrently acquired after each scanning to present the target from different viewpoints. In addition, 3D imaging mode was developed as well in this study: either 3D US or 3D PA images could be reconstructed within 1 minute after the scanning. 

In Figure 8D, 3D US image provides the information of the knurl structure of the subcutaneous tumor, while 3D PA image could delineate a concrete shape of the tumor, as shown in Figure 8E. That is, the profile of this subcutaneous tumor could be clearly observed based on above reconstruction measures. Additionally, two other wavelengths (i.e., 750 nm and 850 nm) were employed in this study to derive HbT and SO_2_ information in real-time. The two wavelengths were directed toward the target alternatively for calculating the HbT and SO_2_, with half of the frame rate used for a single wavelength. The normalized HbT-US co-registered X-Z plane B-scan image is shown in Figure 8F. The distribution of HbT was highly correlated with the subcutaneous tumor profile acquired by US imaging. The normalized SO_2_ image was also acquired by our system, as shown in Figure 8G. This image clearly exhibits lower SO_2_ surrounding subcutaneous tumor tissues, indicating hypoxic tumor vasculature [35]. Note that the yellow and red dashed lines indicate the tumor regions in Figure 8F,G, respectively.

## 4. Discussion and Conclusions

In this study, we have demonstrated how to design/build a handheld, real-time PA imaging system from simulations (i.e., fluence and pressure simulations using COMSOL software and ultrasonic simulation using Vantage software) for applications in neurological diseases such as stroke and tumors. 

FEM simulation is especially powerful for solving the PDE such as the diffusion equation [15,36]. This simulation can handle complex geometries, set proper boundary conditions and has advantages in solving inhomogeneity structures. Our simulation with the diffusion equation can simulate light propagation and distribution in a highly scattering medium, such as human tissues, while geometric optics can help us trace the light path and visualize the effect of the incident angle in water. The Monte Carlo simulation is a common tool and was used for the same purpose in a previous study [14]. The light distribution with the variations of angles and intervals has been investigated using the Monte Carlo simulation. Their results showed that the light fluence dropped less than 7% at a depth of 10 mm when the incident angle changed from 15 degrees to 35 degrees. In addition, at the same depth (i.e., 10 mm), the light fluence decreased more than 25% when the interval between the two fiber arms increased from 10 mm to 14 mm. Their simulation results are consistent with ours (Figure 3B,C), which support our simulation models/works [14]. However, compared with the FEM simulation, the Monte Carlo simulation needs more computational capability to derive the precise solution for the RTE. In addition, a previous study cross-validated the Monte Carlo simulation, FEM and analytical solution methods in solving light transmission in skin. Comparing the Monte Carlo and FEM models, there were transition gaps in the photon density profile when light passes through different layers of skin by using the Monte Carlo simulation, whereas the results of FEM showed smooth transitions between different layers [19]. Another drawback of the Monte Carlo model for simulating the PA effect is that it can be used to assess the optical part only, instead of the entire procedure of PA signal generation. That is, the calculated data must be processed using other tools, such as the k-wave toolbox in MATLAB, to generate the US responses. In contrast, COMSOL can process data from the light distribution to PA signal generation [15,16]. Our simulation model of PA signal generation involves the following modules: PDE, heat transfer, solid mechanics and acoustic generation. Specifically, our model considers not only the fluence diffusion but also the effects of material properties, such as thermal conductivity and Young’s modulus. In addition, for SO_2_ measurement, to compensate for the differences of scattering and absorption coefficients of different wavelengths in soft tissues, we also employed this simulation model to examine the light propagation of 750 nm and 850 nm wavelengths for adjusting the incident light intensity (e.g., the incident laser intensity at 750 nm should be 12.2% larger than at 850 nm to ensure a consistent light intensity at a depth of 10 mm in scattering medium). Overall, we built the PA simulation model using COMSOL in this study to optimize the light path design in the selected medium and evaluate the performance on PA responses [24]. According to the simulation results, we customized the PA probe with an optimized incident angle and interval between the two arms of the fiber bundle, allowing the pump beam to penetrate deeper in both water and the scattering medium. In future studies, we will further cross validate our results (using COMSOL) with other simulation methods (such as Monte Carlo + k-wave) to examine the performance of our simulation models for different applications. Note that the white circular area in the center of each subfigure of Figure 4B is the imaging target. In our study, we did not demonstrate PA generation inside the target due to the module restriction. That is, the normal displacement of the target surface was used as the acoustic pressure source for observation in the medium, and we were unable to assign a boundary condition to the target surface for simultaneously evaluating the PA pressure propagations inside the target and in the medium (i.e., the simulation module disabled the boundary of the target surface when we tried to evaluate both the PA pressure propagations). Thus, we observed only the wave propagation as shown in Figure 4B. 

For in vivo applications, this developed PA imaging system is useful for monitoring disease conditions in multiple disease models. We successfully demonstrated that the symptoms of neurological diseases, such as ischemia during stroke and angiogenesis/hypoxia in a tumor, could be identified by our PA imaging system in real-time (e.g., B-scan image). Additionally, reconstructed MIP, multi-slice C-scan and 3D interactive images could be acquired/displayed after each scanning within a minute, showing the profile of the imaging target from different viewpoints (Figure 8 and Figure S4). As an example, in the near future, the middle cerebral artery occlusion (MCAo) disease model (i.e., the global stroke model) would benefit from this current system because of the deep imaging depth of the PA imaging system with an adequate lateral resolution, allowing for the monitoring of stroke progression. In addition, our high-energy/repetition rate pulsed laser can be employed for biomedical applications based not only on intrinsic contrast agents but on specific extrinsic probes such as nanoparticles. For example, PA imaging using dual-mode rare-earth nanoparticles with different excitation wavelengths could be utilized to almost simultaneously visualize multiple targets [37,38]. Furthermore, our PA imaging system and customized triple-modality nanoparticles with tumor biomarkers could be used for clinical applications in delineating brain tumor margins in the future. 

Moreover, a high-performance US platform (i.e., the Vantage 128 system) was used to achieve real-time PA imaging analysis and display based on its radio frequency data transfer rate via 8 PCI express lanes (up to 6.6 GB/s). Because the data acquisition rate into local memory could be up to 100,000 frames/s, this US platform can also be utilized for ultrahigh-frequency US imaging. That is, we can develop a multimodal system including both PA and ultrafast US imaging to acquire multifaceted information. For example, in brain imaging, the US imaging function of the system could provide information on blood vessel structure and blood flow, while the PA imaging function can provide the status of functional hemodynamics, such as SO_2_, for preclinical examinations. 

In summary, we provide a guide for designing a handheld, real-time PA imaging probe and system for preclinical neurological disease applications. FEM simulations were employed to evaluate the influence of incident angle and interval between the two arms of the fiber bundle for fluence propagation in scattering medium. In addition, a PA wave simulation model was built to assess the design strategy of the PA probe. Ultrasonic simulations were also conducted to determine the actual imaging resolutions of the current US imaging system, and phantom experimental validations were performed to examine the simulation results. The experimental results were consistent with the simulations, indicating that the interval between the two arms of the fiber bundle plays a major role in fluence propagation. Using the designed PA probe based on the given optimal parameters, we successfully reconstructed a color-coded PA image of phantom targets with depth information. Additionally, in vivo PTI and subcutaneous tumor experiments were conducted to verify the performance of the handheld PA imaging system in real-time. In the near future, we will continue to optimize the designs of the fiber bundle with attachable focusing components to achieve a higher energy density to increase the detection sensitivity. For instance, a lens tube will be customized for housing different kinds of optical components, with a specific connector at the end for connecting the fiber bundle. An optical diffuser could be used for the dark-field illumination at a deeper target region, while a cylindrical lens (i.e., to generate the laser line) will be used to design a bright-field handheld probe for delivering the incident light directly under the surface of the US linear-array transducer, increasing the SNR. In addition, we believe that the developed PA model can be employed to design PA nanoparticles for diverse applications, and provide directions for building optical/PA imaging systems to probe neurovascular functions in different disorders using small animal models.

## Supplementary Materials

The following materials are available online at http://www.mdpi.com/1424-8220/18/11/4081/s1, Figure S1: Schematic of the simulation procedures using the FEM method, Figure S2: Schematic of the customized fiber bundle, Figure S3: Schematic of the light propagation in water and the results of simulations, and Figure S4: Reconstructed PA images of the designed phantom consisting of three threads.
