# Supplementary material for: A Handheld Real-Time Photoacoustic Imaging System for Animal Neurological Disease Models: From Simulation to Realization
Source: Sensors (Basel). 2018 Nov 21;18(11):4081. doi: 10.3390/s18114081 (PMC6263979; doi:10.3390/s18114081)
Supplement: Supplementary file 1 [file sensors-18-04081-s001.pdf]

sensors

MDPI

A Handheld Real-time Photoacoustic Imaging System for Animal Neurological Disease Models: From Simulation to Realization

Yu-Hang Liu[1,*], Yu Xu[1,2], Lun-De Liao[3], Kim Chuan Chan[1] and Nitish V. Thakor[1,2,4]

1. Materials and Methods

1.1. Simulation procedures using the FEM method

Our simulation procedures can be divided into two parts (Figure S1): Part 1, Light fluence distribution, which was simulated in the 1% Lipofundin medium with dimensions of 160 mm × 80 mm × 50 mm. As shown in Figure 3A, we placed the observation points at different depths from the surface of the transducer array to evaluate the fluence intensity changes due to the variations of angles and intervals. Because, the RTE has been simplified as the diffusion equation and can be solely calculated using one module, the computational load was not heavy and the model can be calculated in a relatively large scale. Part 2, PA signals generation. The target was placed 10 mm from the surface and the medium was a 1% Lipofundin sphere with radius of 300 μm, while the fluence intensities at 10 mm calculated from Part 1 was the input light source for the PA signals calculation. The PA wave generation needs to couple four different modules and the computational load has been massively increased, so we need to use a fairly small scale for this PA simulation. However, based on this small scale calculation, the results of PA simulation still reflect the changes of the fiber bundle design (*i.e.,* interval and angle). For example, as shown in Figure 4A, PA simulations can help us differentiate the influence from the interval changes of fiber arms on the intensity of PA signals.

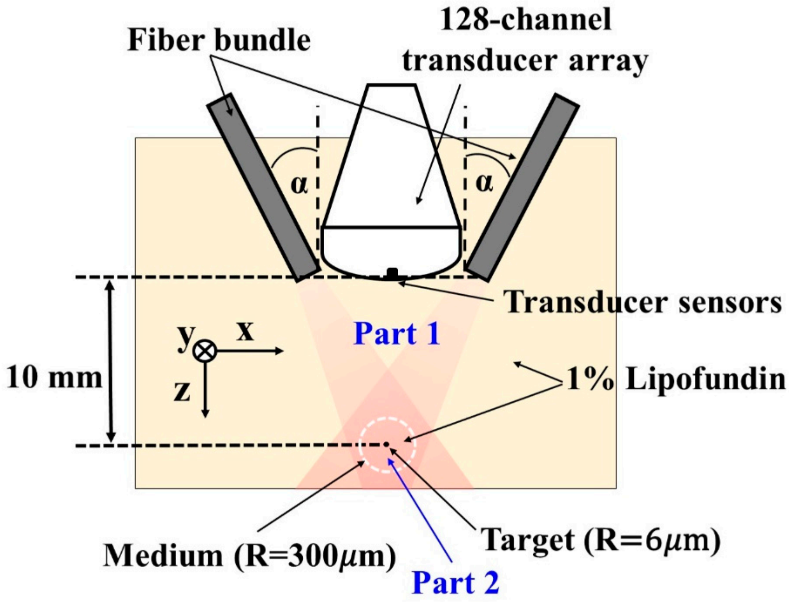

Figure S1. Schematic of the simulation procedures using the FEM method. Light fluence distribution was first conducted to evaluate the fluence intensity changes at different depths due to the variations of angles and intervals. Afterwards, for PA simulation, the target was located 10 mm from the transducer surface, and the results of fluence intensity in scattering medium at a depth of 10 mm (Part 1) were used as the input for the PA wave calculation. Then, to reduce the considerable computation time in this PA simulation model, we conducted our PA simulation on a fairly small scale for evaluation (Part 2).

1.2 Schematic of the customized fiber bundle

In this section, we provided a schematic of the customized fiber bundle. This customized fiber bundle (CeramOptec GmbH, Bonn, Germany) comprises 597 fibers. The input connector of the fiber bundle was specifically designed based on the dimensions of the output socket of the OPO (5 mm in diameter); thus, the fiber bundle can be directly inserted into the OPO to decrease the laser energy loss. The coupling efficiency of the customized fiber bundle is about 65-70%. The fiber bundle has the following outputs: one single fiber was designed with a SubMiniature version A (SMA) connector for monitoring fluctuations in the laser energy; the remaining fibers were evenly distributed into two other arms (as shown in Figure S2 with red and blue colors) with a rectangular output size of 16.5 mm × 0.8 mm.

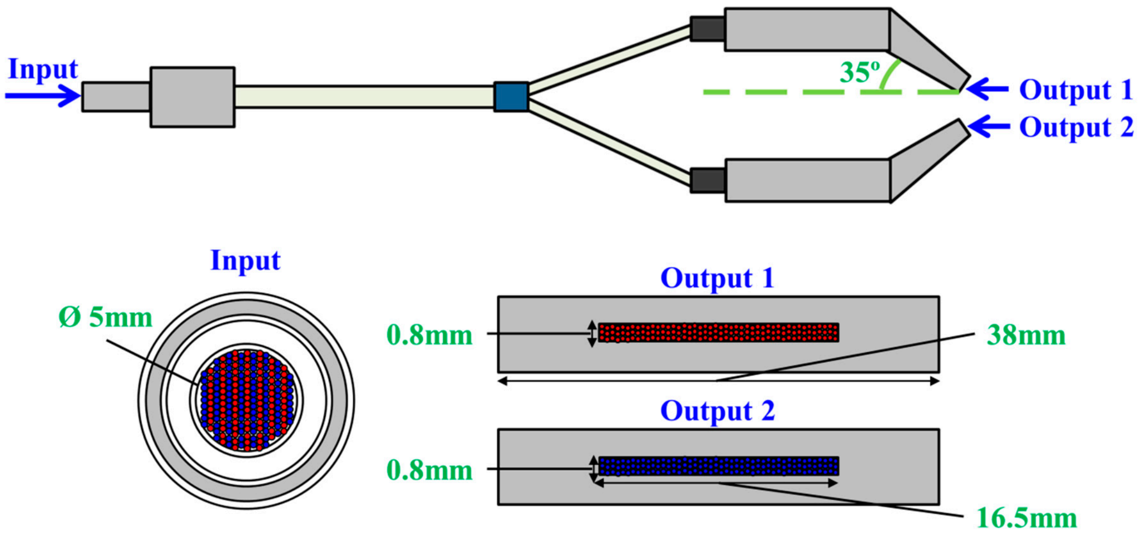

Figure S2. Schematic of the customized fiber bundle. The diameter of the input port is 5 mm, which can be directly connected to the OPO. The 596 fibers were evenly distributed into two arms (*i.e.*, Output 1 and Output 2) for a balanced laser light delivery.

1.3 Light propagation simulation in water

Light propagation in non-scattering medium such as water is common for PA applications, and thus this matter needs to be considered for the simulation as well. However, the precondition for diffusion approximation is that the absorption coefficient of material (μ_a) needs to be much less than the scattering coefficient (μ_s) [1], which is not applicable for water ($\mu_a = 2$ [1/m], $\mu_s = 0.1$ [1/m]) [2, 3]. Therefore, an additional simulation for light propagation in water was employed and conducted in this Supplementary Materials for *in vitro* applications like contrast agent test. The wavelength we used for simulation was 800 nm, while the corresponding absorption coefficient in water is approximately 2 [1/m] [3-5]. Here, using a same wavelength, we focused on the light path design with different incident angles to determine the beam overlapped area covering the target, so the absorption coefficient would not be a major factor for evaluation. In this case, the light transmission in water can be simplified and based on geometric optics, while the numerical aperture of the fiber bundle is 0.22 due to the limitation of manufacturing. The dimensions of the water tank for *in vitro* simulation model were 80 mm × 40 mm × 20 mm. Interval between two fiber arms was fixed at 14 mm, which is the minimum interval we can reach due to the case dimensions of the commercial transducer array. Our strategy for designing the handheld probe is to use one setup for both *in vitro* and *in vivo* applications, and the targeting imaging depth for *in vivo* study is about 10 mm beneath the transducer surface. Therefore, for *in vitro* study simulation, we placed the target at a depth of 8 mm, while light sources were also

put in the water and kept the distance (between the light sources and the bottom surface of water tank) as 10 mm.

2. Results

2.1 Simulation results - light propagation in water

To investigate the effects of incident angle for *in vitro* studies, a geometric optics module was employed (Figure S3A). The simulation results of light transmission in water are shown in Figures S3B-H. At 15 and 25 degrees angles, the two laser beams passed the bottom surface (at a depth of 10 mm) without overlapping each other. For a 35 degrees incident angle, laser beams overlapped from 7 mm deep. At 45 degrees, the overlapped area was between 5 mm and 10 mm in the Z direction. As the incident angle increased, the overlapped area shifted toward the surface (*e.g.,* for 65 degrees, it ranged from 4 mm to 7 mm). When the incident angle came to 75 degrees, the two laser beams overlapped nearly at a depth of 4 mm. Based on the conditions for designing the PA probe (*i.e.,* the light sources and transducer array at the same surface plane), the incident angles from 35 degrees to 45 degrees would provide quality results for *in vitro* studies when the target is placed at a depth of 8 to 10 mm. To integrate the results of both *in vivo* and *in vitro* experiments, 35 degrees and a 14-mm interval would be applicable for both *in vivo* and *in vitro* studies.

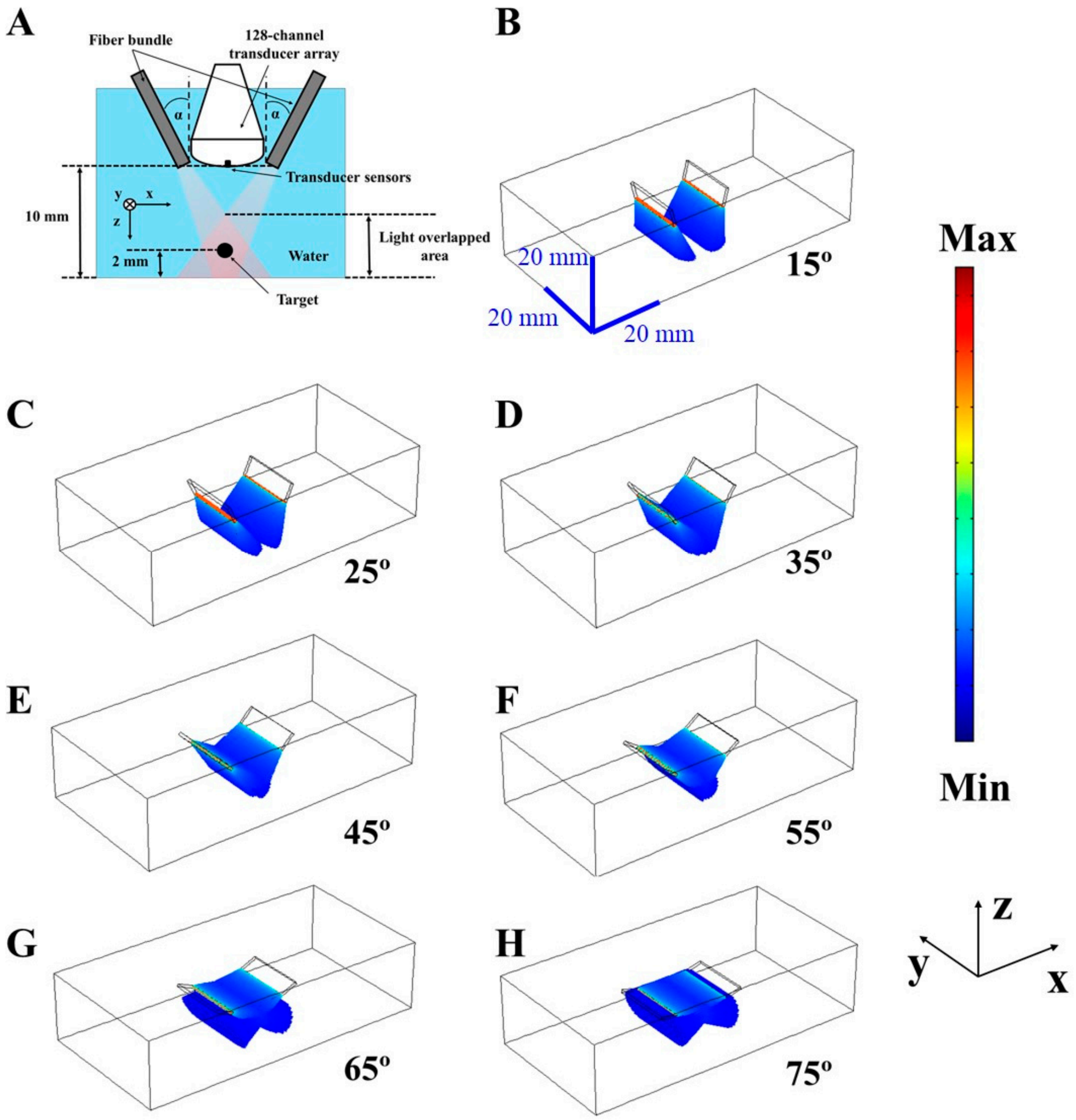

Figure S3. Schematic of the light propagation in water and the results of simulations. (**A**) The configuration of light propagation in water for evaluating the light overlapped area with different incident angles. Similar to Figure 3A, a 128-channel transducer array was placed in the middle of the two arms of the fiber bundle, and the incident angles of the fiber bundle were α. The interval was defined as 14 mm for this *in vitro* evaluation, and the numerical aperture of the fibers was 0.22. The imaging target was placed 8 mm from the transducer surface. (**B**-**H**) The light propagation paths with different incident angles in water (from 15 degrees to 75 degrees). The XYZ scale bars are shown in (**B**), which are applicable to (**C**-**H**) as well. The color bar shows the fluence density.

2.2 Real-time PA imaging reconstruction of a designed phantom

To demonstrate the capability of real-time reconstruction of our PA imaging system, a phantom target consisting three black threads (~500 μm in diameter) for mimicking *in vivo* blood vessels were designed in this paper, as shown in Figure S4A. The entire phantom was placed in water to assess the performance of our PA system. The pump laser light had a wavelength of 750 nm and the energy per pulse was 2 mJ. The scanning stage was used to scan the entire phantom for 20 mm in length with a 50-μm step size in the Y direction to acquire multiple X-Z plane real-time B-scan images. We were able to acquire the 3D reconstructed PA image (Figure S4B) within 1 minute after each scanning. On the other hand, TopView (Figure S4C) and SideView (Figure S4D) MIP PA images of the phantom could be simultaneously acquired within 5 seconds after each scanning, due to a lower computation load compared to 3D reconstruction. Above results showed the capability of our PA imaging system for the real-time evaluation.

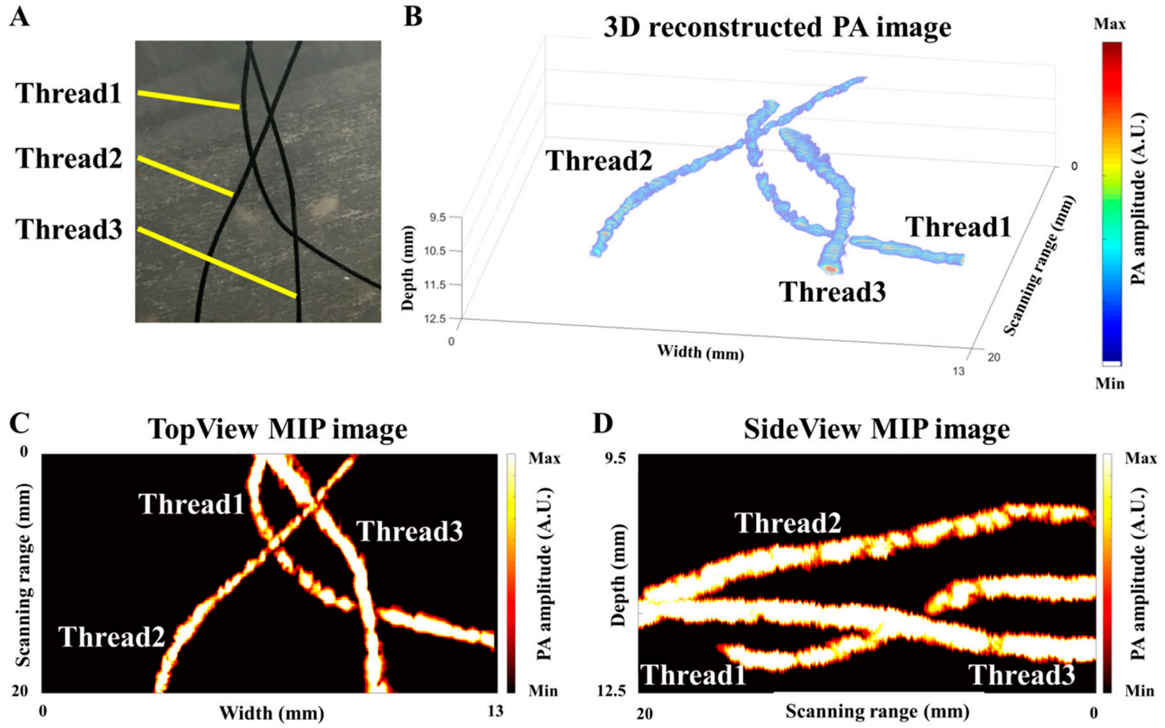

Figure S4. Reconstructed PA images of the designed phantom consisting of three threads. (**A**) Photo of the designed phantom consisting three black threads, which was placed in water for the experiment. (**B**) Reconstructed 3D PA image of the phantom. (**C**) TopView (X-Y plane) MIP PA image of the designed phantom. (**D**) SideView (Y-Z plane) MIP image of the designed phantom. Note that the images only show the depths from 9.5 mm to 12.5 mm in Z-direction.

References

1. Wang, L. V.; Wu, H.-i., *Biomedical optics: principles and imaging*. John Wiley & Sons: 2012.
2. Wang, Z.; Ha, S.; Kim, K., A new design of light illumination scheme for deep tissue photoacoustic imaging. *Opt. Express* **2012,** 20, (20), 22649-22659.
3. Smith, R. C.; Baker, K. S., Optical properties of the clearest natural waters (200-800 nm). *Appl. Opt.* **1981,** 20, (2), 177-84.
4. Hale, G. M.; Querry, M. R., Optical Constants of Water in the 200-nm to 200-microm Wavelength Region. *Appl. Opt.* **1973,** 12, (3), 555-63.
5. Anguita, D.; Brizzolara, D.; Ghio, A.; Parodi, G. In *Smart plankton: a nature inspired underwater wireless sensor network*, Natural Computation, 2008. ICNC'08. Fourth International Conference on Natural Computation, Shandong, China, 18-20 Oct, 2008; IEEE: Shandong, China, 2008; pp 701-705.
